# Adequate antibody response to BioNTech COVID vaccine in a multiple sclerosis patient treated with siponimod

**DOI:** 10.1186/s41983-021-00428-8

**Published:** 2021-12-14

**Authors:** Gulnaz Siddiqui, Heidi Maloni, Victor E. Nava

**Affiliations:** 1 Department of Biomedical Sciences and Department of Pathology, University of Missouri, Kansas City, MO USA; 2 Department of Neurology, Department of Veterans Affairs Medical Center, Washington, DC USA; 3 Department of Pathology, Department of Veterans Affairs Medical Center, Washington, DC USA

**Keywords:** COVID-19, Multiple sclerosis, Anti-COVID-19 vaccination, Sphingosine 1-phosphate receptor, Modulators, Disease-modifying therapy

To the editor,

We appreciated Mansoor and colleagues review entitled “COVID-19 pandemic and the risk of infection in multiple sclerosis patients on disease-modifying therapies: “what the bleep do we know?”” [1]. The authors examined available evidence guiding the management of multiple sclerosis (MS) patients during this pandemic, indicating that sphingosine 1-phosphate receptor modulators (S1PRM), including siponimod, could increase the risk of COVID-19 infection due to immunosuppression.

However, emerging data suggest that MS patients mount a humoral and cellular immune response even while receiving disease-modifying therapies (DMT) [2, 3]. For instance, retrospective data [2] from MS patients receiving S1PRM who completed two doses of anti-SARS-CoV-2 vaccination (either Pfizer or Moderna) showed positive anti-spike (S) protein antibody titers (Abbott or Roche SARS-CoV-2 IgG assay) determined forty-five and half days (average) after immunization. A wide range (16.1–80.4) of IgG index was observed. The incidence of COVID-19 infection, however, was not analyzed [2]. Surprisingly, the study suggested that based on “real-life experience”, S1PRM could potentially hamper an effective humoral response to anti-COVID-19 vaccination in MS patients, which may unnecessarily discourage urgent immunization efforts.

At the *Veterans Affairs Medical Center, Washington DC*, we have followed a 73-year-old man with active progressive MS (Expanded Disability Status Scale of 5.0) since 1995. He was initially treated in 1998 with *glatiramer acetate*, which was switched to siponimod (2 mg daily orally) since December 2019, achieving stabilization of neurocognitive decline. His absolute lymphocyte count was decreased (0.3 K/cmm, reference range 0.8–3.1 K/cmm) since July 2020. He received two doses of the BNT162b2 Pfizer vaccine (2/1/2021 and 2/22/2021), and his quarterly laboratory work-up (*complete cell blood counts, serum immunoglobulins, thyroid function, liver function, Chem 7, urine analysis, and fecal occult blood*) has been unremarkable, except for stable mild lymphopenia (0.3 K/cmm, 7/26/21) and mildly decreased IgM of 36 mg/dl (reference range 43–279 mg/dl). Additional immune evaluation (including lymphocyte subsets) was not performed. Anti-COVID-19 antibody testing was performed about 160 days after completion of vaccination (6/28/2021) and was positive for S (42.3 U/ml units, *Eclisys, Roche*) and negative for anti-nucleocapsid (0 U/ml*, Eclisys, Roche*) proteins, indicating adequate immune response to vaccination and absence of prior SARS-CoV-2 infection.

Universal consensus on anti-COVID-19 vaccination in MS patients treated with DMT is still emerging [2]. PubMed does not yield real-life data on the use of siponimod in MS patients exposed to COVID-19 or vaccination against it. Diminished immune response to non-COVID-19 vaccines have been reported after treatment with siponimod [4], which may be less immunosuppressive than fingolimod (another S1RP inhibitor). Caution is necessary while using DMT in MS [1–3]. One must also consider that many studies are limited to static analysis of humoral responses without correlates with cell blood counts or neutralizing activity [2]. Further research is necessary to determine if DMT hamper appropriate immune responses, especially since the BNT162b2 vaccine also elicits T-cell immunity [5]. In summary, our experience suggests that siponimod may not significantly alter humoral immunity against COVID-19 vaccination, and may contribute to encourage vaccination against this pandemic in MS patients receiving DMT.

## Data Availability

The data sets supporting the conclusion of this article are included within the article.

## Abbreviations

COVID-19: Coronavirus disease 2019
MS: Multiple sclerosis
S1PRM: Sphingosine 1-phosphate receptor modulators
DMT: Disease-modifying therapies
SARS-CoV-2: Severe acute respiratory syndrome coronavirus 2
Chem-7: Basic metabolic panel
BNT162b2: Pfizer-BioNTech COVID-19 vaccine
